# Imprinting on time-structured acoustic stimuli in ducklings

**DOI:** 10.1098/rsbl.2021.0381

**Published:** 2021-09-29

**Authors:** Tiago Monteiro, Tom Hart, Alex Kacelnik

**Affiliations:** Department of Zoology, University of Oxford, Oxford OX1 3PS, UK

**Keywords:** acoustic stimuli, *Anas platyrhynchos*, filial imprinting, mallard, innate pre-dispositions, behavioural timing

## Abstract

Filial imprinting is a dedicated learning process that lacks explicit reinforcement. The phenomenon itself is narrowly heritably canalized, but its content, the representation of the parental object, reflects the circumstances of the newborn. Imprinting has recently been shown to be even more subtle and complex than previously envisaged, since ducklings and chicks are now known to select and represent for later generalization abstract conceptual properties of the objects they perceive as neonates, including movement pattern, heterogeneity and inter-component relationships of same or different. Here, we investigate day-old Mallard (*Anas platyrhynchos*) ducklings’ bias towards imprinting on acoustic stimuli made from mallards’ vocalizations as opposed to white noise, whether they imprint on the temporal structure of brief acoustic stimuli of either kind, and whether they generalize timing information across the two sounds. Our data are consistent with a strong innate preference for natural sounds, but do not reliably establish sensitivity to temporal relations. This fits with the view that imprinting includes the establishment of representations of both primary percepts and selective abstract properties of their early perceptual input, meshing together genetically transmitted prior pre-dispositions with active selection and processing of the perceptual input.

## 1. Background

Newborn nidifugous birds such as ducks and chickens quickly learn to identify their mother and follow her around for protection, warmth and foraging information, a phenomenon known as filial imprinting [1].

As a learning mechanism, imprinting is notable because it lacks explicit (observable) reinforcement [2] and exposes nature and nurture in one sweep; the mechanism itself is inherited and narrowly pre-specified, but the content of what is learned reflects the circumstances of the newborn. Research has shown that (i) sensory pre-dispositions are crucial, facilitating neonates' orientation towards relevant features of the environment [3,4] and that (ii) abstract concepts can be acquired by ducklings through imprinting [5]. These discoveries suggest that imprinted target representations are better treated as multidimensional vectors of perceptual objects' affordances than as libraries of sensory percepts [6]. This novel interpretation makes biological sense, considering that successful algorithms for identifying a suitable target must be robust with respect to scale, perspective and shape while exploiting all available information to infer identity [1,7,8]. Here, we investigate the potential roles played by predisposed sound preferences and sensitivity to the temporal structure.

We may expect precocial newborns to use their sensory input to form a ‘concept’ of the parental object that will serve as a target for following. The sensory dimensions that may be expected to participate in such concepts should of course be defined and constrained by the ‘umwelt’ of the organism [9], which must include temporal information. Neuroscientists have identified neural codes for spatial (morphological) properties of perceived objects [10], but the encoding of data along the fourth dimension, time, is relatively less well known [11,12]. For this reason, exploring how temporal features of sensory input participate in the representation of imprinting objects should enrich understanding of how imprinting works, while adding evidence for how fundamental is the ability to keep track of events in time (see also [13]).

**Figure 1.**
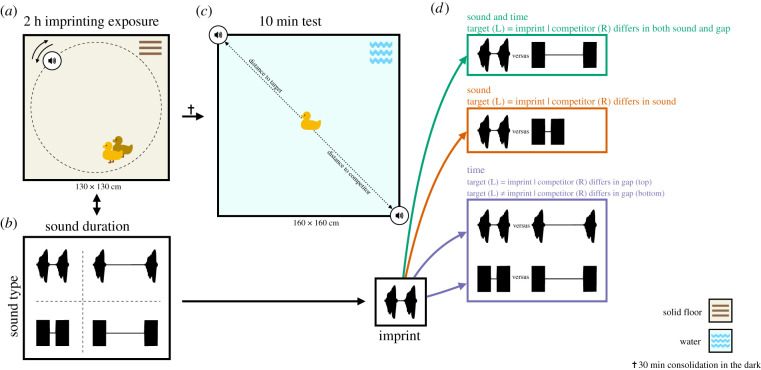
Experimental environment. (*a*). Training set-up: pairs of ducklings were exposed for 2 h to a single compound stimulus, broadcast from a speaker revolving around the arena. (*b*) Compound sound stimuli (normalized amplitude traces) varied along with two dimensions, sound type and duration of a silent gap between two brief sound bursts. Sounds bursts could be composed of snippets of duck sound or white noise bursts (rows), separated by silent gaps lasting either 0.2 or 1.2 s, thus creating compounds of either 1 or 2 s total duration (columns). (*c*) Testing set-up: following a 30 min consolidating period in the dark, ducklings were tested individually in a water pool with speakers at fixed locations, broadcasting two different stimuli compounds. (*d*) Example of testing conditions for a particular imprinting exposure stimulus (see Extended figure S1 in the electronic supplementary material for a complete depiction of all testing conditions). Across conditions, targets were the same as the imprint, except in the last ‘time’ condition (time, bottom example), when the ducks were tested for preference between two stimuli made up of a sound novel to them. Depending on the testing condition, competitor stimuli could differ from the target in both sound and gap duration (sound and time); only sound (sound); only gap duration (‘time’, top example); or again only gap duration, but with both target and competitor made up with novel sounds (‘time’, bottom example).

Drawing inspiration from psychophysical timing protocols [14,15], we exposed day-old ducklings to acoustic compound stimuli that varied in the period of silence between two sounds (calls) (figure 1*a*). To take into account innate pre-dispositions [16], the calls were either made from duck vocalizations or white noise bursts, depending on treatment (figure 1*b*). Focusing on the duration of silent gaps rather than duration of sounds controls for differences in amount of sound energy, thus isolating sensitivity to temporal structure.

## 2. Methods

### (a) Subjects

Two hundred and eighty-four domesticated mallard ducklings (*Anas platyrhynchos domesticus*) of unknown sex were supplied by *Foster's Poultry*, Gloucestershire, as eggs, and returned to the supplier as young birds after participating in the experiments. The eggs were assigned to four imprinting groups and four test conditions, resulting in 16 subgroups. The group size was the result of finding a compromise between the numbers used in the previous studies (e.g. [5]) and our drive to reduce the number of experimental animals, as part of our compliance to the 3Rs. Group sizes (minimum 12, maximum 20 individuals) were affected by hatching rates.

### (b) Incubation and hatching

Eggs were incubated for 25 days in a *Brinsea Ova-Easy 190* incubator at 37.7°C and 40% humidity and moved to a *Brinsea Ova-Easy* hatcher for the last 3 days of incubation at higher humidity regimes (65% or more). Hatching took place in the dark and ducklings remained in the hatching chamber for 12–24 h, as the peak sensitive period is between 13 and 40 h of age [17].

### (c) Experimental design

The four imprinting groups resulted from a factorial design combining two sound types and two silent gap durations. Stimuli were made out of either two snippets from a female duck vocalization or two white noise bursts (all 0.4 s long), separated by *short* (0.2 s) or *long* (1.2 s) silent gaps (figure 1*b*). Total stimulus duration was thus 1 or 2 s, respectively. Preference tests (see figure 1*c,d* for examples and electronic supplementary material, Extended figure S1 for full experimental design), contrasted a *target* and a *competitor* stimulus. The *target* shared the duration of the silent gap with the imprinted stimulus (called the ‘imprint’ below) but between calls of either the same or the alternative sound type, and at test time the *competitor* differed from the target in either both dimensions (sound type and gap duration) or only one of them. Tests thus offered a choice between (i) a target identical to the imprint and a competitor differing in both sound and gap duration; (ii) a target identical to the imprint and a competitor made of the alternative sound but sharing the original gap duration; (iii) a target identical to the imprint and a competitor made out of the original sound but with the alternative gap duration; and (iv) a target made of the alternative sound and the original gap duration against a competitor made of the alternative sound and the alternative gap duration. The discriminant dimensions in the four conditions were, respectively: sound and time, sound only, time only and again time only but between novel sounds.

### (d) Stimulus design

Stimuli were generated using *Audacity* software (audacityteam.org). The duck sound was made by extracting a 0.4 s snippet of female adult mallard vocalization from the Macaulay Library at the Cornell Lab of Ornithology audio file (ML 133222). Session-long sequences were compiled into mp3 files using custom *Matlab* code (2020a, Mathworks). Variable inter-stimulus intervals were drawn from a normal distribution (*u* = 15, *σ* = 5 s).

### (e) Imprinting exposure

Following previous protocols [5,18], we combined priming that enhances imprinting responses in chickens and ducklings [19,20] with 2 h-long imprinting exposure periods. Pairs of ducklings (pairing reduces stress) were exposed in an arena (130 × 130 cm, figure 1*a*) to a revolving wireless speaker (*EasyAcc*, model LX-839) repeatedly playing the corresponding imprinting stimulus (see figure 1*b*, electronic supplementary material, Extended figure S1, and Experimental design, above). The speaker was suspended 15 cm above the floor by a thin fishing line attached to a revolving boom. Each revolution lasted approximately 40 s, with a diameter of 1 m, as the movement has been shown to enhance imprinting [21]. Following imprinting exposure, and Bateson's (1966) protocol [1], duckling pairs were placed in a dark chamber for a 30 min retention interval.

### (f) Testing

Individual ducklings were tested for 10 min in a water pool (180 × 180 cm, figure 1*c*), with two test stimuli. The combination of imprinting exposure on a dry arena with testing on water mimics natural circumstances for imprinting and locomotory mode during ulterior following responses. One of the two stimuli (the ‘target’) always shared the gap duration of the imprint, but could be made of the same (test conditions (i), (ii) and (iii)) or the alternative sound (test condition (iv)), while a ‘competitor’ differed in either only sound (test condition (ii)), only silent gap (test condition (iii)) or both (test conditions (i) and (iv), see figure 1*d*). Test stimuli were placed in two diametrically opposed fixed locations (position of target and competitor stimuli balanced across subjects). The combination of four imprinting and four testing conditions means that there were 16 subgroups (figure 1*d*; electronic supplementary material, Video S1 and Extended figure S1).

### (g) Data acquisition, processing and analyses

The video was recorded using *Sony* wireless 4 K action cameras (FDR-X3000 R) at 30 Hz. A colour thresholding method was implemented using custom *Bonsai* code [22] to track the position of the duckling and each speaker. Position data were downsampled to 1 Hz, rotated and normalized relative to the position of the speakers using custom *Matlab* code (2020a, Mathworks), so that speakers were always at the x,y positions [−1,0] and [1,0] (see electronic supplementary material, Extended figure S2). The first 20 s of each test were discarded to allow the duckling to get used to being in water for the first time. We then computed the second-by-second Euclidean distance in pixels between the duckling and each of the speakers (schematics in figure 1*b*). For every animal, we computed a preference index ‘*Delta*’ by subtracting the average Euclidean difference for the *competitor* speaker from the average Euclidean distance to the *target* one, so that positive *Deltas* correspond to *target* and negative ones to *competitor* preference (−2 ≤ *Delta* ≤ 2). Violin plots included in the electronic supplementary material, Extended figure S3, were created using [23].

We used a three-way ANOVA with *Delta* (i.e. preference) as a response variable and the following three categorical input variables: imprinting sound (two levels: duck sound or white noise), imprinting silent gap duration (two levels: short or long) and test condition (four levels: sound, gap duration, both and gap duration between novel sounds), and all pairwise interactions to test for preference (R aov function):

$$\begin{array}{rl} & Preference\;∼\;Imprint\;Sound\;Type\\ & \;+Imprint\;Silent\;Gap\;Duration\\ & \;+Test\;Condition+[all\;pairwise\;interactions]. \end{array}$$

The interaction plot shown in the electronic supplementary material, Extended figure S4 was built using R function (cat_plot); for details see: https://www.rdocumentation.org/packages/interactions/versions/1.1.3/topics/cat_plot

## 3. Results

The overall experimental design is summarized for reference in figure 1. Please refer to figure 1*d* for testing examples, and electronic supplementary material, Extended figure S1 for full description. Note that we define the stimulus that each duck experienced during the imprinting exposure as the ‘imprint’ and to the stimulus that individuals would choose if expressing a preference for the relevant tested variable as the ‘target’. The *target* was the same as the *imprint*, except in one of the ‘time’ conditions, when ducks were tested for preference between two stimuli made up of a sound novel to them.

**Figure 2.**
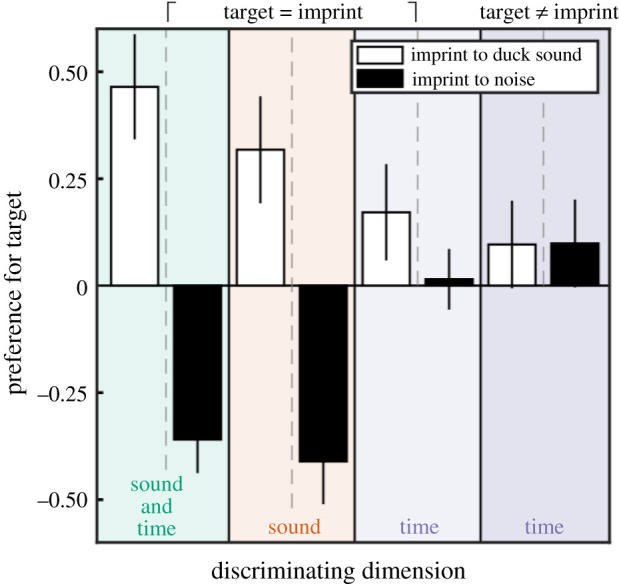
Preference in tests. Preference index (means ± s.e.m.; *n* = 284, see main text for details) as a function of imprinting exposure sound (duck sound in white, white noise in black) and discriminant dimension in testing condition (from left to right, sound and time, sound only, time between familiar sounds and time between novel sounds). The same colour scheme as in figure 1.

Figure 2 (see electronic supplementary material, Extended figure S3 for individual animal data) shows that on average ducklings preferred the stimulus constructed with a natural duck call (white bars) over that constructed of white noise regardless of which one was experienced in the earlier imprinting phase. In the two time-testing conditions, when the sound did not serve as discriminant, but only the duration of the silent gap could identify the *target*, they displayed a weak overall bias for the *target*, as would be expected from imprinting on temporal structure, but this bias does not reach statistical reliability across the different sound compositions. This suggests that there may be some sensitivity to the temporal relation that would require further testing, but kind of sound is likely to play a major role.

The interaction between *sound type* used during imprinting exposure and *test condition* (electronic supplementary material, Extended figure S4) was highly significant (*F*_3,271_ = 8.049, *p* < 0.001; figure 2), as was the main effect of sound type (*F*_1,271_ = 29.473, *p* < 0.001), but not that of temporal structure. In particular, figure 2 shows that, overall, ducklings did show a weak bias towards the gap duration experienced during imprinting, but the trend is vulnerable to both sound type and the requirement to generalize to novel sounds at test time, hence cannot be considered to be proven.

## 4. Discussion and conclusion

Overall our results point to the complexity of information processing during imprinting. In particular, they do not reliably establish whether ducks imprint on the temporal relation between acoustic stimuli and/or if they generalize to temporal intervals between acoustic stimuli across sound types. In a condition testing preference between an imprinted stimulus and a competitor differing in both sound type and temporal gap, ducklings systematically approached the test stimulus made of duck vocalizations, even when this meant rejecting their previously experienced duration. In the two testing conditions when only time duration could serve as discriminant, they showed a weak bias towards the imprinted duration, even when tested with novel sounds, but these results can at the moment only be considered to be suggestive and worthy of further investigation. With hindsight, it is possible to identify reasons why, even if time properties of the imprinting stimuli did play a role in forming the parental concept, our tests may have failed to demonstrate it reliably. First, we asked for discriminations between durations of a silent gap between sounds, rather than imprinting and testing with duration of sounds. We chose duration of silences because this controls for non-temporal factors such as the amount of acoustic energy, and because it highlights the connection with relational concept imprinting rather than perceptual imprinting, but it is clearly a more difficult task. Second, we generated stimuli by sampling natural vocalizations and white noise, and found the strongest temporal responses when using the former. This is consistent with an attentional interpretation: if ducklings pay more attention to sounds with the power spectrum and other physical features typical of their species, then they may also be secondarily more sensitive to time features simply because they attend more closely to such signals. Third, the durations between which our ducklings were asked to discriminate silent gaps were 0.2 and 1.2 s. These durations were chosen because given their substantial ratio (1 : 6) they should be discriminable, but we have no *a priori* knowledge of the absolute range of durations to which neonate ducks show greater sensitivity.

In summary, ducklings clearly imprint more easily on sounds with the properties of duck vocalizations than white noise, but further evidence must be collected to reliably establish whether they also imprint on the temporal relation between sounds. Overall, this confirms the appeal of envisaging imprinting as the establishing of representations of parental targets constituted by concept vectors with multiple attributes. Such a process articulates innate pre-dispositions with sensitivity to experience and perceptual data with abstract relations. The dimensions of such vectorial representations are only starting to be mapped.

## Data accessibility

All data can be found as electronic supplementary material and at https://datadryad.org/stash/share/hCdF2WhcsjUsxUXia2Kb8vTq53aItP8-O8OyV2m2C4I.

The code that supports the findings of this study is available at https://github.com/PTMonteiro/MonteiroHartKacelnik_2021.git

The data are provided in the electronic supplementary material [24].
